# Effect of health foods on cytochrome P450-mediated drug metabolism

**DOI:** 10.1186/s40780-017-0083-x

**Published:** 2017-05-10

**Authors:** Takamitsu Sasaki, Yu Sato, Takeshi Kumagai, Kouichi Yoshinari, Kiyoshi Nagata

**Affiliations:** 1 Department of Environmental and Health Science, School of Pharmaceutical Sciences, Tohoku Medical and Pharmaceutical University, 4-4-1 Komatsushima, Aoba-ku, Sendai, Miyagi 981-8558 Japan; 2 Department of Molecular Toxicology, School of Pharmaceutical Sciences, University of Shizuoka, 52-1 Yada, Suruga-ku, Shizuoka 422-8526 Japan

**Keywords:** Health food, Health food-drug interaction, Cytochrome P450, Inhibition

## Abstract

**Background:**

Health foods have been widely sold and consumed in Japan. There has been an increase in reports of adverse effects in association with the expanding health food market. While health food-drug interactions are a particular concern from the viewpoint of safe and effective use of health foods, information regarding such interactions is limited owing to the lack of established methods to assess the effects of health food products on drug metabolism. We therefore developed cells that mimicked the activities of cytochrome P450 1A2 (CYP1A2), CYP2C9, CYP2C19, CYP2D6, and CYP3A4, which strongly contribute to drug metabolism in human hepatocytes, and established a system to assess the inhibitory activity of health foods toward P450-mediated metabolism.

**Methods:**

We simultaneously infected HepG2 cells with five P450-expressing adenoviruses (Ad-CYP1A2, Ad-CYP2C9, Ad-CYP2C19, Ad-CYP2D6, and Ad-CYP3A4) to mimic the activity levels of these P450s in human hepatocytes, and named them Ad-P450 cells. The activity levels of P450s in Ad-P450 cells and human hepatocytes were calculated via simultaneous liquid chromatography/tandem mass spectrometry analysis utilizing a P450 substrate cocktail.

**Results:**

We established Ad-P450 cells mimicking the activity levels of CYP1A2, CYP2C9, CYP2C19, CYP2D6, and CYP3A4 in human hepatocytes. We determined the Km values of P450 substrates and IC_50_ values of P450 inhibitors in Ad-P450 cells. These values were approximately equivalent to those obtained in previous studies. We investigated the inhibitory effects of 172 health foods that were recently in circulation in Japan on P450-mediated metabolism using Ad-P450 cells. Of the 172 health foods, five products (two products having dietary effects, one turmeric-based product, one collagen-based product, and one propolis-containing product) simultaneously inhibited the five P450s by more than 50%. Another 29 products were also confirmed to inhibit one or more P450s.

**Conclusions:**

We established a comprehensive assessment system to elucidate the effects of health foods on P450-mediated metabolism and identified the inhibitory activity of 34 of 172 health foods toward the drug-metabolizing P450s. Our results may provide useful information to predict health food-drug interactions.

## Background

Foods with health claims and so-called ‘health foods’ have been widely sold and consumed in Japan. Among these foods, Foods for Specified Health Uses (FOSHU) have shown scientific evidence-based beneficial effects on physiological conditions in both healthy and diseased individuals. Although most health foods contain ingredients that promote health and improve health-related conditions, the effectiveness of these products has not been proven. Consumers therefore select products based on advertisements, which are usually non-scientific. However, the consumption of health foods has increased rapidly, because they are inexpensive and readily available in comparison with FOSHU. Reporting of adverse effects has also increased in association with the expansion of the health food market [1]. Several studies have reported that drug-induced liver injury is caused not only by prescription drugs but also by dietary and herbal supplements [2, 3]. Consumers have recently become more aware of the benefits and risks of using health foods, through information provided by relevant regulatory agencies. However, many consumers believe that products derived from natural substances are harmless. Furthermore, some consumers use multiple health foods at the same time. Provision of information that is accompanied by scientific evidence on the safety and efficacy of health foods is essential to maintain public health and decrease medical expenses.

Health food-drug interactions are a particular concern from the viewpoint of safe and effective use of health foods. Common interactions are associated with the inhibition of drug-metabolizing enzymes, particularly cytochrome P450s (CYPs, P450s). For example, green tea extract has been reported to inhibit CYP2C9, CYP2D6, and CYP3A4 in human liver microsomes [4]. Epigallocatechin gallate, the most abundant catechin in green tee, is also a potent inhibitor of CYP3A in human liver and intestinal microsomes [5]. While ginkgolide A, B, C, J, and bilobalide, known as constituents of *Ginkgo biloba*, have shown weak or negligible inhibition of CYP1A2, CYP2C9, and CYP3A in human liver microsomes, other constituents, such as ginkgolic acid I and II, have been reported to inhibit CYP1A2, CYP2C9, and CYP2C19 [6, 7]. Although many reports have provided useful information on the safe and effective use of health foods in patients taking drugs, most of this information is limited to the effects of the health foods main ingredients on P450-mediated metabolism. However, health foods that are on the market in Japan contain a number of ingredients, and such products may contain impurities. We recently reported the effects of health foods that are available in Japan on CYP2D6-mediated metabolism [8]. We have confirmed that a product containing curcumin is a potent inhibitor of CYP2D6. *Coleus forskohlii* extract- and collagen-based products also inhibit CYP2D6. The inhibition by forskolin and collagen had not been previously reported. Thus, it is difficult to deduce the effects of health foods on P450-mediated metabolism solely from the assessment of their main ingredients. We therefore believe that the development of a P450 inhibition screening system for complete health food products, rather than their main ingredients, may lead to more appropriate use of the products.

Human hepatocytes are recommended as the most reliable tool for the assessment of drug metabolism and drug-drug interactions [9]. However, due to their high cost and lot-to-lot variations in drug metabolism, it is difficult to continuously obtain human primary hepatocytes that have the same metabolic activities, and therefore these cells are unsuitable for high-throughput testing. In this study, we have utilized hepatocellular carcinoma cells (HepG2 cells) and P450-expressing adenoviruses to establish cells (named Ad-P450 cells) that mimic the activities of CYP1A2, CYP2C9, CYP2C19, CYP2D6, and CYP3A4, which strongly contribute to human drug metabolism in human hepatocytes [10]. We have established a system to assess the inhibitory effects of health foods on P450-mediated metabolism using Ad-P450 cells.

## Methods

### Reagents

Phenacetin, acetaminophen, dextromethorphan, dextrorphan, furafyllin, and sulfaphenazole were purchased from Sigma-Aldrich (St. Louis, MO, USA). Quinidine was purchased from Tokyo Chemical Industry (Tokyo, Japan). 4-hydroxydiclofenac and 1-hydroxymidazolam were purchased from Becton Dickinson (Franklin Lakes, NJ, USA). 5-Hydroxyomeprazole was purchased from Toronto Research Chemical (North York, Canada). Ketoconazole was purchased from LKT Laboratories (St. Paul, MN, USA). Health foods were purchased from Japanese retail markets. All other reagents were of the highest grade available from Wako Pure Chemical Industries (Osaka, Japan) and Sigma-Aldrich. Oligonucleotides were commercially synthesized by Fasmac (Atsugi, Japan).

### Construction of recombinant adenovirus

The open reading frames of *CYP1A2*, *CYP2C9*, *CYP2C19*, and *CYP3A4* were amplified by PCR from cDNA obtained from human hepatocytes by using primers specific for CYP1A2 (forward: 5′-CACCATGGCATTGTCCCAGTCTGTTC-3′; reverse: 5′-TCAGTTGATGGAGAAGCGCAGCCG-3′), CYP2C9 (forward: 5′-CACCATGGATTCTCTTGTGGTCCTTG-3′; reverse: 5′-TCAGACAGGAATGAAGCACAGCTGGTAG-3′), CYP2C19 (forward: 5′-CACCATGGATCCTTTTGTGGTCCTTGTG-3′; reverse: 5′-TCAGACAGGAATGAAGCACAGCTGA-3′), and CYP3A4 (forward: 5′-CACCATGGCTCTCATCCCAGACTTGGC-3′; reverse: 5′-TCAGGCTCCACTTACGGTGCCATC-3′), respectively. Constructs of these P450-expressing adenoviruses, Ad-CYP1A2, Ad-CYP2C9, Ad-CYP2C19, and Ad-CYP3A4, were made according to the procedure described [11]. Preparation of Ad-CYP2D6 was previously described [8].

### Human hepatocytes and cell culture

Human cryopreserved primary hepatocytes (lot HEP187265, 54-year-old Caucasian woman) were purchased from Biopredic International (Rennes, France). The hepatocytes were thawed and cultured using the medium kit (Biopredic International) according to the manufacturer’s protocol. The cells were seeded in type I collagen-coated 48-well plate at 8.5 × 10^4^ cells/well. After 12 h, the cell medium was changed with culture medium (Biopredic International) containing P450 substrate cocktail (100 μM phenacetin, 25 μM diclofenac, 10 μM omeprazole, 10 μM dextromethorphan, and 10 μM midazolam) and then the cells were incubated for 24 h.

### Preparation of HepG2 cells mimicking the activity levels of CYP1A2, CYP2C9, CYP2C19, CYP2D6, and CYP3A4 in human hepatocytes (Ad-P450 cells)

HepG2 cells were purchased from RIKEN cell Bank (Tsukuba, Japan) and were cultured in Dulbecco’s modified Eagle’s medium (Wako Pure Chemical Industries) supplemented with 10% fetal bovine serum (Biowest, Miami, FL, USA), non-essential amino acids (Thermo Fisher Scientific, Maltham, MA, USA), and antibiotic-antimycotic (Thermo Fisher Scientific) under 5% CO_2_-95% air at 37 °C. The cells were seeded in a 48-well tissue culture plate (Becton Dickinson) at 5.0 × 10^4^ cells/well. After 48 h, the cells were simultaneously infected with Ad-CYP1A2 at 5 multiplicity of infection (MOI), Ad-CYP2C9 at 1 MOI, Ad-CYP2C19 at 2 MOI, Ad-CYP2D6 at 0.05 MOI, and Ad-CYP3A4 at 10 MOI. The cells were cultured for 72 h and subsequently used as Ad-P450 cells. The cells were incubated in culture medium containing P450 substrate cocktail for 5 h.

### Assessment of P450 activity and inhibition in Ad-P450 cells

Ad-P450 cells were incubated in culture medium containing phenacetin (2.5–100 μM), diclofenac (0.5–20 μM), omeprazole (0.25–10 μM), dextromethorphan (1–50 μM), or midazolam (0.25–10 μM) for 5 h. In P450 inhibition assessments, Ad-P450 cells were incubated in culture medium containing P450 substrate cocktail and each typical P450 inhibitors (furafylline for CYP1A2 [0.05–30 μM], sulfaphenazole for CYP2C9 [0.01–10 μM], ticlopidine for CYP2C19 [0.3–300 μM], quinidine for CYP2D6 [0.01–10 μM], or ketoconazole for CYP3A4 [0.01–10 μM]) for 5 h.

### Preparation of health food extracts and curcumin

Recommended daily dose of health foods (tablet, capsule, and powder) was incubated in 10 mL of 70% ethanol for 2 h at 37 °C. The resulting solution was centrifuged at 3500 × g for 15 min and the supernatant was used as a test solution. Liquid-type health foods were used as test solutions without extraction procedure. Curcumin was dissolved in dimethyl sulfoxide (DMSO).

### Effect of health foods and curcumin on P450-mediated metabolism

Ad-P450 cells were incubated in culture medium containing P450 substrate cocktail and test solution (0.5%) or curcumin (10–300 μM) for 5 h.

### Liquid chromatography/tandem mass spectrometry (LC-MS/MS) analysis

Collected medium was mixed with an equal volume of ethyl acetate containing 0.1 μM nitrazepam (internal standard). After shaking, the mixtures were centrifuged at 2500 × g for 10 min and the supernatants were evaporated to dryness at 60 °C in a block incubator. The residues were dissolved in acetonitrile containing 0.1% acetic acid and the solutions were subjected to liquid chromatography/tandem mass spectrometry (LC-MS/MS) analysis.

The Prominence system (Shimadzu Corporation, Kyoto, Japan) equipped with LCMS-8040 system (Shimadzu Corporation) was used for LC-MS/MS analysis with an electrospray ionization interface. The ionization mode used was positive in the multiple reaction monitoring. The chromatographic separation was performed on Xterra MS C18 columns (2.1 mm × 100 mm, 5 μm) (Waters, Milford, MA, USA). The column oven temperature was maintained at 40 °C. The mobile phase consisted of acetonitrile containing 0.1% acetic acid and water containing 0.1% acetic acid (85:15) with a flow rate of 200 μL/min. The LC-MS/MS conditions were shown in Table 1. Desolvation line temperature and heat block temperature was 250 and 400 °C, respectively. Nebulizer gas flow rate and drying gas flow rate were 3 and 15 L/min, respectively.

**Table 1:** MS/MS selected reaction monitoring transitions and collision energies for ions of metabolite and internal standard in the cocktail assays

	CYP1A2	CYP2C9	CYP2C19	CYP2D6	CYP3A4	IS
Substrate	Phenacetin	Diclofenac	Omeprazole	Dextromethorphan	Midazolam	Nitrazepam
Metabolite	Acetaminophen	4′-Hydroxy-diclofenac	5-Hydroxy-omeprazole	Dextrorphan	1′-Hydroxy-midazolam	-
Collision energy (eV)	19	33	13	42	23	25
Product ion (m/z)	110.05	229.95	213.95	157.05	324.05	236.10

### Data analysis

The Km, Vmax, and IC_50_ values were determined using Prism software (version 6.0; GraphPad Software Inc., San Diego, CA, USA). The other calculations were performed using Excel (Microsoft, Seattle, MA, USA).

## Results

### Establishment of Ad-P450 cells

**Fig. 1:**
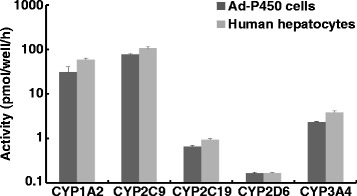
Activity levels of CYP1A2, CYP2C9, CYP2C19, CYP2D6, and CYP3A4 in Ad-P450 cells and human hepatocytes. Five human P450s were expressed in HepG2 cells as described in section titled ‘Materials and methods’. The cells (Ad-P450 cells) were cultured for 72 h and then incubated in culture medium containing P450 substrate cocktail for 5 h. Human cryopreserved primary hepatocytes seeded in type I collagen-coated 48-well plate at a density of 8.5 × 10^4^ cells/well were incubated with culture medium containing P450 substrate cocktail for 24 h. These media were collected and metabolites were analyzed by LC-MS/MS. The activity levels are shown as means ± SD (*n* = 3)

The activity levels of CYP1A2 (phenacetin *O*-deethylation activity), CYP2C9 (diclofenac 4′-hydroxylation activity), CYP2C19 (omeprazole 5-hydroxylation activity), CYP2D6 (dextromethorphan *O*-demethylation activity), and CYP3A4 (midazolam 1′-hydroxylation activity) in human hepatocytes were calculated via simultaneous LC-MS/MS analysis utilizing the P450 substrate cocktail. The activities of CYP1A2, CYP2C9, CYP2C19, CYP2D6, and CYP3A4 were 59.48, 106.71, 0.94, 0.17, and 3.86 pmol/well/h, respectively (Fig. 1). Based on these data, we simultaneously infected HepG2 cells with Ad-CYP1A2 (5 MOI), Ad-CYP2C9 (1 MOI), Ad-CYP2C19 (2 MOI), Ad-CYP2D6 (0.05 MOI), and Ad-CYP3A4 (10 MOI) to mimic the activity levels of CYP1A2, CYP2C9, CYP2C19, CYP2D6, and CYP3A4 in human hepatocytes. The activities of CYP1A2, CYP2C9, CYP2C19, CYP2D6, and CYP3A4 in the infected HepG2 cells were 31.22, 77.02, 0.66, 0.16, and 2.33 pmol/well/h, respectively (Fig. 1). Although the P450 activities in HepG2 cells infected with five P450-expressing adenoviruses were slightly lower in comparison with those in human hepatocytes, the ratios of the activities of each P450 were almost identical between these two cells (Fig. 1). We thus named the established HepG2 cells mimicking the activity levels of CYP1A2, CYP2C9, CYP2C19, CYP2D6, and CYP3A4 in human hepatocytes, Ad-P450 cells.

### Kinetic analysis and inhibition assessment in Ad-P450 cells

**Fig. 2:**
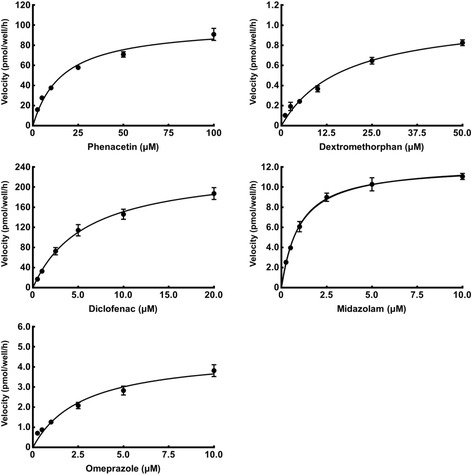
Kinetic analysis of P450-mediated metabolism in Ad-P450 cells. Ad-P450 cells were incubated in culture medium containing phenacetin (2.5–100 μM), diclofenac (0.5–20 μM), omeprazole (0.25–10 μM), dextromethorphan (1–50 μM), or midazolam (0.25–10 μM) for 5 h. These media were collected and metabolites were analyzed by LC-MS/MS. The activity levels are shown as means ± SD (*n* = 3)

**Fig. 3:**
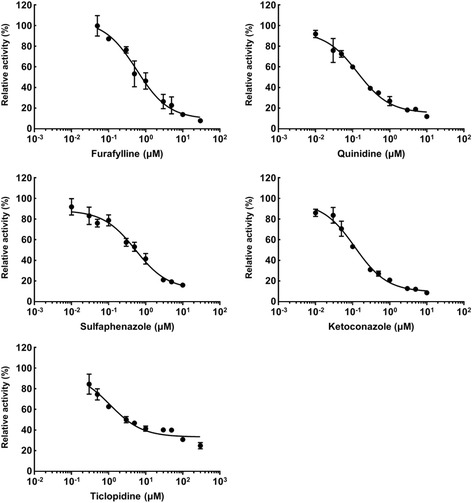
Effect of representative inhibitors on P450-mediated metabolism in Ad-P450 cells. Ad-P450 cells were incubated in culture medium containing P450 substrate cocktail and each typical P450 inhibitor (furafylline [0.05–30 μM], sulfaphenazole [0.01–10 μM], ticlopidine [0.3–300 μM], quinidine [0.01–10 μM], or ketoconazole [0.01–10 μM]) for 5 h. These media were collected and metabolites were analyzed by LC-MS/MS. The activity levels are shown as means ± SD (*n* = 3)

To investigate the properties of Ad-P450 cells, we performed kinetic analyses using P450 substrates and calculated IC_50_ values for specific representative P450 inhibitors (CYP1A2, furafylline; CYP2C9, sulfaphenazole; CYP2C19, ticlopidine; CYP2D6, quinidine; CYP3A4, ketoconazole) in Ad-P450 cells (Figs. 2 and 3). The Michaelis-Menten equation was used to calculate the Km and Vmax values from the metabolic reaction rates of the five P450s (Table 2). The specific P450 inhibitors used showed concentration-dependent inhibition, and the IC_50_ values are shown in Table 2. These values are similar to those previously reported [12, 13]. These results suggest that Ad-P450 cells are useful for the assessment of P450-mediated drug metabolism, and drug-drug or health food-drug interactions.

**Table 2:** Kinetic parameters of P450-mediated drug metabolism and IC_50_ values of P450 inhibitors in Ad-P450 cells

Enzyme	Metabolic reaction	Inhibitor	Km (μM)	Vmax (pmol/well/hr)	IC_50_ (μM)
CYP1A2	Phenacetin *O*-deethylation	Furafylline	16.32	100.30	0.57
CYP2C9	Diclofenac 4′-hydroxylation	Sulfaphenazole	5.87	239.9	0.53
CYP2C19	Omeprazole 5-hydroxylation	Ticlopidine	2.75	4.66	1.11
CYP2D6	Dextromethorphan *O*-demethylation	Quinidine	17.14	1.10	0.14
CYP3A4	Midazolam 1′-hydroxylation	Ketoconazole	1.00	12.30	0.12

### Effects of health foods on P450-mediated metabolism

**Fig. 4:**
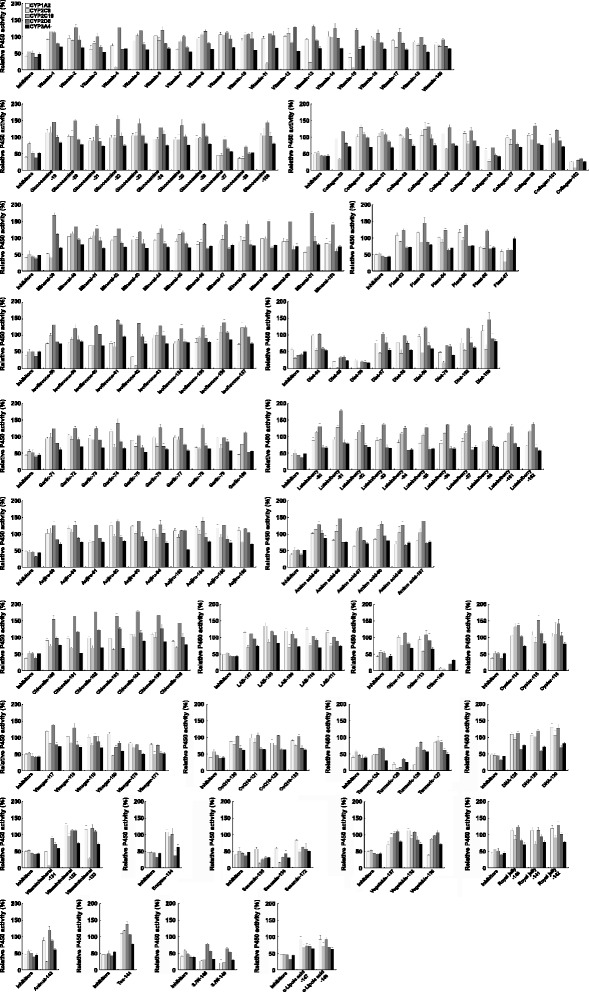
Effect of health foods on P450-mediated metabolism in Ad-P450 cells. Ad-P450 cells were incubated in culture medium containing P450 substrate cocktail and test solution (0.5%) for 5 h. These media were collected and metabolites were analyzed by LC-MS/MS. The P450s activities in Ad-P450 cells cultured in P450 substrate cocktail and 0.5% extractant (70% ethanol) for 5 h were set to 100%. The activity levels are shown as means ± SD (*n* = 3)

We investigated the inhibitory effects of 172 health foods, whose uses in Japan were confirmed in our previous survey [8], on P450-mediated metabolism in Ad-P450 cells. The results, classified by main ingredients or expected effects, are shown in Fig. 4 and are summarized in Table 3. Products that inhibited any P450 by more than 50% were considered to have P450 inhibitory activity.

**Table 3:** Health foods with inhibitory activity and residual activity of P450s

Product classification	Number^a^	Product name	Inhibited P450	Relative activity (%)^b^				
				CYP1A2	CYP2C9	CYP2C19	CYP2D6	CYP3A4
Vitamin	4/19	Vitamin-4	CYP2C9	73.0	8.6	―	62.8	64.0
		Vitamin-11	CYP2C9	95.1	18.1	―	―	65.4
		Vitamin-13	CYP2C9	92.4	23.3	―	88.1	65.3
		Vitamin-15	CYP1A2, CYP2C9	38.2	7.3	―	62.6	72.6
Glucosamine	2/11	Glucosamine-27	CYP1A2	45.1	83.6	92.4	65.6	56.2
		Glucosamine-28	CYP1A2	36.4	70.0	70.2	50.7	53.2
Collagen	3/12	Collagen-29	CYP2C9	96.1	31.8	―	81.4	71.4
		Collagen-36	CYP2C9, CYP2D6, CYP3A4	61.4	27.7	69.1	46.2	42.2
		Collagen-152	CYP1A2, CYP2C9, CYP2C19, CYP2D6, CYP3A4	26.3	8.0	30.4	35.6	26.6
Mineral	1/14	Mineral-39	CYP2C9	52.3	41.5	―	―	70.1
Plant	1/6	Plant-57	CYP2C9	58.1	28.4	63.6	63.5	98.3
Isoflavone	1/10	Isoflavone-62	CYP1A2, CYP2C9	35.7	8.5	―	97.4	75.0
Diet	6/9	Diet-65	CYP1A2, CYP2C9, CYP2C19, CYP2D6, CYP3A4	22.3	3.8	32.4	34.7	23.9
		Diet-66	CYP1A2, CYP2C9, CYP2C19, CYP2D6, CYP3A4	24.2	3.4	20.4	19.7	17.1
		Diet-67	CYP2C9	76.5	44.3	―	77.6	55.1
		Diet-68	CYP2C9	77.4	46.3	99.1	75.8	55.3
		Diet-69	CYP2C9	94.7	48.5	―	68.2	60.0
		Diet-70	CYP1A2, CYP2C9, CYP3A4	49.1	19.0	68.4	65.3	40.2
Garlic	1/10	Garlic-160	CYP1A2	45.2	77.3	―	53.4	56.3
Lutein/berry	0/11	―	―	―	―	―	―	―
Aojiru	0/10	―	―	―	―	―	―	―
Amino acid	0/6	―	―	―	―	―	―	―
Chlorella	0/7	―	―	―	―	―	―	―
Lactic acid bacterium	0/5	―	―	―	―	―	―	―
Other	1/3	Other-168	CYP1A2, CYP2C9, CYP2C19, CYP2D6, CYP3A4	7.6	5.3	0.0	20.9	32.5
Oyster	0/3	―	―	―	―	―	―	―
Vinegar	1/6	Vinegar-169	CYP2C9	―	46.4	70.8	83.9	59.6
CoQ10	0/4	―	―	―	―	―	―	―
Turmeric	3/4	Turmeric-124	CYP1A2, CYP2C9, CYP3A4	47.2	49.4	67.6	66.4	30.2
		Turmeric-125	CYP1A2, CYP2C9, CYP2C19, CYP2D6, CYP3A4	19.2	5.6	10.2	35.4	25.1
		Turmeric-126	CYP1A2	18.3	70.9	85.7	61.8	57.8
DHA	0/3	―	―	―	―	―	―	―
Vitamin/mineral	2/3	Vitamin/mineral-131	CYP1A2, CYP2C9	49.2	7.3	89.6	71.7	59.0
		Vitamin/mineral-133	CYP2C9	―	28.4	―	―	71.5
Enzyme	1/1	Enzyme-134	CYP2D6	―	94.6	―	36.6	62.5
Sesamin	3/3	Sesamin-135	CYP2C9, CYP2C19, CYP2D6, CYP3A4	56.8	12.5	25.6	31.5	31.4
		Sesamin-136	CYP2C9, CYP2C19, CYP2D6, CYP3A4	58.5	15.7	32.4	43.4	31.0
		Sesamin-172	CYP2C9	83.0	49.4	62.8	57.3	51.1
Vegetable	1/3	Vegetable-139	CYP1A2	36.8	86.8	97.4	―	71.5
Royal jelly	0/3	―	―	―	―	―	―	―
Animal	1/1	Animal-143	CYP2C9	88.5	24.6	―	88.6	61.5
Tea	0/1	―	―	―	―	―	―	―
SJW	2/2	SJW-145	CYP1A2, CYP2C9, CYP3A4	26.5	31.3	78.0	57.0	32.5
		SJW-146	CYP1A2, CYP2C9, CYP3A4	21.4	22.3	64.3	53.8	29.9
α-Lipoic acid	0/2	―	―	―	―	―	―	―

^a^Number of products with inhibitory activity/Number of products examined

^b^P450s that were not inhibited by products are shown as ―

Of the 172 products, five products [two products having dietary effects (diet, no. 65 and 66), one turmeric-based product (no. 125), one collagen-based product (no. 152), and one other product (propolis-containing product, no. 168)] simultaneously inhibited the five P450s by more than 50%. Two sesamin-based products (no. 135 and 136) simultaneously inhibited the four P450s except CYP1A2. Five products [collagen-based (no. 36), diet (no. 70), turmeric-based (no. 124), St. John’s wort (SJW)-based (no. 145 and 146)] simultaneously inhibited three P450s (CYP1A2, CYP2C9/CYP2D6, and CYP3A4). A vitamin-, an isoflavone, and a vitamin/mineral-based product (no. 15, 62, and 131) simultaneously inhibited two P450s (CYP1A2 and CYP2C9). In addition, nineteen other products were found to inhibit one of the five P450s. Therefore, our results demonstrated that 34 (19.8%) of 172 health foods have P450 inhibitory activities.

### Effects of curcumin on P450-mediated metabolism

**Fig. 5:**
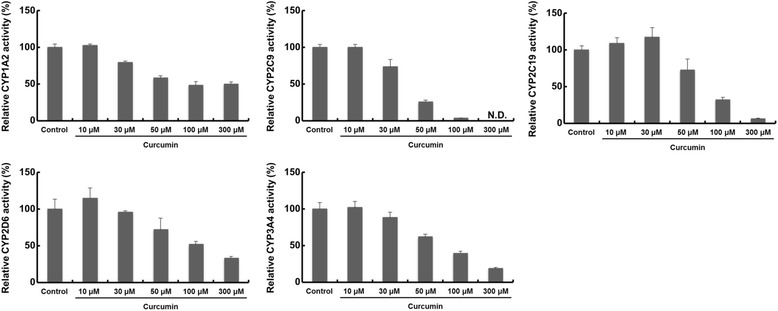
Effect of curcumin on P450-mediated metabolism in Ad-P450 cells. Ad-P450 cells were incubated in culture medium containing P450 substrate cocktail and curcumin (10–300 μM) for 5 h. These media were collected and metabolites were analyzed by LC-MS/MS. The P450 activities in Ad-P450 cells cultured in P450 substrate cocktail and DMSO (0.5%) for 5 h were set to 100%. The activity levels are shown as means ± SD (*n* = 3). The limit of quantitation is 18.90 ng/mL for acetaminophen, 39.02 ng/mL for 4′-hydroxydiclofenac, 0.24 ng/mL for 5-hydroxyomeprazole, 0.25 ng/mL for dextrorphan, and 2.14 ng/mL for 1′-hydroxymidazolam. N.D.: not detected

Of the five products that simultaneously inhibited the five P450s, we further examined the effects of their ingredients in the turmeric-based products on P450-mediated metabolism. Curcumin is a known constituent of turmeric and a P450 inhibitor [14]. In our system using Ad-P450 cells, curcumin inhibited the five P450s in a concentration-dependent manner (Fig. 5). The residual activities in Ad-P450 cells treated with 50 μM curcumin were 58.3% for CYP1A2, 25.9% for CYP2C9, 72.7% for CYP2C19, 71.9% for CYP2D6, and 61.9% for CYP3A4. Similar to the results of turmeric-based products, CYP2C9 was most strongly inhibited by curcumin, although this inhibition was observed only at a relatively high concentration.

## Discussion

In order to comprehensively elucidate health food-drug interactions, we developed Ad-P450 cells mimicking the activity levels of CYP1A2, CYP2C9, CYP2C19, CYP2D6, and CYP3A4 in human hepatocytes and established a P450 inhibition assessment system. Of the 172 health food products tested, five products simultaneously inhibited all the five P450s, and another 29 products inhibited at least one of the P450s. Furthermore, the results of the inhibition of CYP2D6 in Ad-P450 cells were consistent with those obtained using Ad-CYP2D6-infected cells in our previous study [8].

To develop Ad-P450 cells that mimicked the drug-metabolizing activity of human hepatocytes, the five P450s that most strongly contribute to drug metabolism in human livers were expressed in HepG2 cells at levels showing the same activity as those of human hepatocytes (Fig. 1). We determined the Km values for typical P450 reactions and IC_50_ values of representative P450 inhibitors in the Ad-P450 cells. These values were approximately equivalent to those obtained in previous studies (Figs. 2 and 3, Table 2) [12, 13]. These results show that the Ad-P450 cells are useful tools to assess drug metabolism and health food-drug interactions.

Based on the data from our previous survey [8], we investigated the effects of health foods, for which actual use in Japan has been confirmed, on the five P450s. Our results showed that five products (two diet products, one turmeric-based product, one collagen-based product, and one propolis-containing product) inhibited the five P450s by more than 50% (Fig. 4). The two diet products (no. 65 and 66) contained *C. forskohlii* extract powder (containing 10% forskolin). However, there are few reports concerning their P450 inhibitory activities. These results suggest that unidentified ingredients might be involved in P450 inhibition, since these two diet products do not include common ingredients other than *C. forskohlii* extract.

A turmeric-based product (no. 125) also inhibited the five P450s, with CYP2C9 being the most inhibited. Curcumin is a polyphenolic component in turmeric that inhibits CYP1A2, CYP2B6, CYP2C9, CYP2D6, and CYP3A4, and its IC_50_ values are particularly low for CYP2C9 [14]. Since the product was obtained from *Curcuma longa*, which is curcumin-rich, and strong inhibitory activity of other turmeric-based products (no. 124 and 126) with little/no *C. longa* had not been observed, the inhibition of the five P450s by the turmeric-based product was thought to be due to curcumin. In this study, we confirmed that curcumin inhibited the five P450s in a concentration-dependent manner in Ad-P450 cells (Fig. 5). However, strong inhibition was found only at relatively high concentrations. These results suggest that P450 inhibition by the turmeric-based product could also be associated with ingredients other than curcumin.

Based on the product labels, the collagen-based product (no. 152) that showed inhibitory activities toward the five P450s contained silibinin in addition to collagen, but other collagen-based products (no. 29–38 and 151) did not. Silibinin has been reported to inhibit CYP2C9 and CYP3A4 through mechanism-based inhibition (MBI) [15]. It was recently reported that a metabolite of rutaecarpine, a principal constituent of *Evodia rutaecarpa*, strongly inhibits P450s [16], suggesting that health food-drug interactions could be caused by P450 inhibition through MBI.

We also revealed that two sesamin-based products (no. 135 and 136) inhibited CYP2C9, CYP2C19, CYP2D6, and CYP3A4. Sesamin is a known competitive inhibitor of CYP1A2, CYP2C9, and CYP3A4 and the reported Ki values are 75, 24, and 4.2 μM, respectively [17]. Our results support the previous study, since the inhibition of CYP1A2 by two sesamin-based products was weaker than that of other P450s.

We confirmed that one isoflavone-based (no. 62) and one vitamin-based (no. 15) product inhibited CYP1A2 and CYP2C9. The isoflavone-based product contained isoflavones derived from soybean and red clover, such as genistein and biochanin A, which reportedly inhibit CYP1A2 [18, 19]. In contrast, few reports are available on P450 inhibition by vitamins in vitamin-based products, although lipid-soluble vitamins, such as vitamin A and vitamin D, have been reported to induce CYPs [20, 21]. Based on the product label, the vitamin-based product (no. 15) does not contain vitamin A and vitamin D, while the amounts of water-soluble vitamins, such as vitamins B1, B2, B6, B12, and pantothenic acid, were higher than those in other vitamin-based products. Although further study is needed to elucidate the P450 inhibition by vitamin-based product, the use of excessive amounts of water-soluble vitamins should be avoided to prevent health food-drug interactions.

In this study, we established a system to assess the inhibitory effects of health foods on P450-mediated metabolism using Ad-P450 cells. In contrast to the assessment of health food-drug interactions using human hepatocytes, assessment using Ad-P450 cells might not provide an accurate prediction of the interaction because of the limited number of P450s expressed in Ad-P450 cells. However, this established assessment system is easily applicable to health food-drug interactions testing for many health foods, because Ad-P450 cells are inexpensive with little to no lot-to-lot variations. In comparison with common assessment systems of health food-drug interactions using liver microsomes, our established system is including absorption process of chemical compounds in health foods into cells [11]. Moreover, this system has a valuable advantage, where it can mimic hepatocytes of P450-mediated metabolism, which showed interindividual variations by adjusting the ration of infection amount and species of P450-expressing adenovirus [22]. Furthermore, it is of great importance to assess the effects of health foods on the five P450s that most strongly contribute to drug metabolism in human livers in order to obtain beneficial and fundamental information under the current situation where there is limited scientific evidence regarding health food-drug interactions.

## Conclusions

We established a comprehensive assessment system to investigate the effects of health foods on P450-mediated metabolism and found that 34 of the 172 health foods have the potential to inhibit human P450 activities. This report is the first to investigate the P450 inhibitory effects of a large number of health foods under the same conditions. Our results provide useful information to understand and predict health food-drug interactions.

## Abbreviations

Ad: Adenovirus
FOSHU: Foods for Specified Health Uses
LC-MS/MS: Liquid chromatography/tandem mass spectrometry
MBI: Mechanism-based inhibition
MOI: Multiplicity of infection
PCR: Polymerase chain reaction
